# Computational Investigations on the Natural Small Molecule as an Inhibitor of Programmed Death Ligand 1 for Cancer Immunotherapy

**DOI:** 10.3390/life12050659

**Published:** 2022-04-29

**Authors:** Geethu S Kumar, Mahmoud Moustafa, Amaresh Kumar Sahoo, Petr Malý, Shiv Bharadwaj

**Affiliations:** 1 Department of Life Science, School of Basic Science and Research, Sharda University, Greater Noida 201310, Uttar Pradesh, India; geethusaji1440@gmail.com; 2 Center for Bioinformatics, Computational and Systems Biology, Pathfinder Research and Training Foundation, Greater Noida 201308, Uttar Pradesh, India; 3 Department of Biology, Faculty of Science, King Khalid University, Abha 62529, Saudi Arabia; mfmostfa@kku.edu.sa; 4 Department of Botany and Microbiology, Faculty of Science, South Valley University, Qena 83523, Egypt; 5 Department of Applied Sciences, Indian Institute of Information Technology Allahabad, Allahabad 211015, Uttar Pradesh, India; 6 Laboratory of Ligand Engineering, Institute of Biotechnology of the Czech Academy of Sciences, v.v.i., BIOCEV Research Center, 25250 Vestec, Czech Republic

**Keywords:** programmed death ligand 1, natural products, immunotherapy, Neoenactin B1, molecular dynamics simulation

## Abstract

Several therapeutic monoclonal antibodies approved by the FDA are available against the PD-1/PD-L1 (programmed death 1/programmed death ligand 1) immune checkpoint axis, which has been an unprecedented success in cancer treatment. However, existing therapeutics against PD-L1, including small molecule inhibitors, have certain drawbacks such as high cost and drug resistance that challenge the currently available anti-PD-L1 therapy. Therefore, this study presents the screening of 32,552 compounds from the Natural Product Atlas database against PD-L1, including three steps of structure-based virtual screening followed by binding free energy to refine the ideal conformation of potent PD-L1 inhibitors. Subsequently, five natural compounds, i.e., Neoenactin B1, Actinofuranone I, Cosmosporin, Ganocapenoid A, and 3-[3-hydroxy-4-(3-methylbut-2-enyl)phenyl]-5-(4-hydroxybenzyl)-4-methyldihydrofuran-2(3H)-one, were collected based on the ADMET (absorption, distribution, metabolism, excretion, and toxicity) profiling and binding free energy (>−60 kcal/mol) for further computational investigation in comparison to co-crystallized ligand, i.e., JQT inhibitor. Based on interaction mapping, explicit 100 ns molecular dynamics simulation, and end-point binding free energy calculations, the selected natural compounds were marked for substantial stability with PD-L1 via intermolecular interactions (hydrogen and hydrophobic) with essential residues in comparison to the JQT inhibitor. Collectively, the calculated results advocate the selected natural compounds as the putative potent inhibitors of PD-L1 and, therefore, can be considered for further development of PD-L1 immune checkpoint inhibitors in cancer immunotherapy.

## 1. Introduction

Cancer is a severe concern faced by researchers worldwide due to the increase in the number of patients and its capacity to shorten the human lifespan. The International Agency for Research on Cancer (IARC) estimated ~19.3 million new cancer cases and almost 10 million cancer deaths in their Global Cancer Statistics (GLOBOCAN) 2020 report, in which one-half of the new cases and more than 58% of cancer deaths occurred in Asian countries [1]. Although conventional treatment strategies, surgery, radiotherapies, and chemotherapy have shown massive progress in treating cancer within the past centuries, cancer patients still endure problems during treatment due to the low efficacy and side effects of conventional therapeutics [2,3,4]. For example, multidrug resistance in cancer, which leads to the reduction in the efficacy rate of treatment and slows down the chances of finding a cure, has been linked with several mechanisms, including enhanced drug efflux, genetic mutations, tumor microenvironment (TEM), certain growth factors, and an increase in the metabolic rate of xenobiotics [2,5,6,7,8]. Likewise, the efficacy of cancer radiotherapy, which mainly targets tumor cells and minimally damages the normal cells, is based on the acceptable dose tolerance capacity of the adjacent normal cells and associated with post-therapy session side effects, such as skin peeling, blister formation, itching, fatigue, soreness, dry mouth, and even hair loss [9]. Thus, with a profound understanding of the disease and the drawbacks of the therapeutic strategies, an innovative approach is required for cancer treatment, which mainly focuses on the specific target cells without damaging the adjacent healthy cell [10].

In the tumor microenvironment (TEM), cancer cells power the activation of various immune checkpoint pathways that trigger immune suppression. Thus, cancer immunotherapy, which aims to stimulate the immune system’s ability to fight cancer, is in high demand in cancer treatment [11,12,13]. The advantage of this therapy is that it can prevent metastasis and recurrence of cancer cells and destroy primary cancer, marked as a standard treatment for cancer patients [14]. Programmed cell death 1 (PD-1, also known as CD279) T cell receptor and its ligands—programmed death ligand 1 (PD-L1 or B7-H1 or CD274) and programmed death ligand 2 (PD-L2 or B7-DC or CD273)—are the prime immune checkpoint pathway components, which are targeted for immune checkpoint inhibitory therapy against cancer [13,15]. 

Programmed death ligand 1 (PD-L1) is a glycoprotein of 290 amino acids mainly expressed on the surface of cardiac endothelium, placenta, pancreatic isles, immune cells, epithelial cells, and tumor cells apart from T cells, B cells, and antigen-presenting cells (APC) [16,17,18]. The various cancer cells express PD-L1 and utilize the PD-1/PD-L1 signaling pathway as an escape route from the T cell autoimmunity to promote tumor growth [19]. For instance, the binding of PD-L1 to its receptor, i.e., PD-1, leads to suppression of T cell migration, proliferation, and secretion of cytokines and hinders the ability of T cells to destroy tumor cells [20]. Thus, the various inhibitors of PD-1 and PD-L1, which can reverse T cell suppression and enhance the anti-tumor immune responses in cancer patients, have been reported [20,21]. For example, atezolizumab, avelumab, and durvalumab monoclonal antibodies (mAbs) are the FDA approved inhibitors that target PD-L1 to terminate the tumor progression [20]. These therapeutics are highly effective to restore T cell-mediated anti-tumor immunity and showed unprecedented success in cancer therapy [22]. However, these therapeutics are associated with several insufficiencies; for instance, antibodies are obstructed from invasion into tumors due to their large size [23], which may contribute to a partial antagonizing effect on PD-1/PD-L1 signaling at the anticipated therapeutic site, and resulting in suboptimal efficacy against tumors [24]. Additionally, these antibodies have been reported for immune-related adverse events (irAEs), involving autoimmune hepatitis, colitis, and pneumonitis [25,26]. An additional limitation of antibodies is the development of resistance in tumors, durability, and high production costs, creating the need of introducing new small molecules as potential inhibitors [5,27,28]. From this perspective, small-molecule based inhibitors are being researched to intervene in the PD-1/PD-L1 signaling pathway in a fight against cancer, recently reviewed elsewhere [29,30].

Recent reports have also studied the bioactive compounds derived from natural resources to exhibit anti-cancer properties by suppressing the gene expression responsible for tumor progression, making them easy to access and cost-effective [31,32,33]. For instance, 

S-Allylcysteine, an organo-sulfur, and Silibinin (a natural compound derived from *Silybum marianum* or milk thistle) are examples of natural compounds known to act as potential PD-L1 inhibitors [34,35]. Even though targeting the interface of PD-1/PD-L1 by small molecule inhibitors is challenging due to its 3D geometry, i.e., large and flat binding pockets, constant efforts have been added in the discovery of small molecules as PD-L1 inhibitors and reflected by the number of growing publications and patents [36,37]. However, none of the reported small molecules have progressed into clinical trials to date [38]. Thus, finding natural products with the ability to inhibit PD-L1 can lead to the development of a novel small molecule-based cancer immunotherapy. Additionally, molecular docking simulations were recently applied to identify the potential terphenyl-based small-molecule inhibitors against PD-L1 protein to disturb the PD-1/PD-L1 signaling pathway [38]. Therefore, in this study, we have utilized the multi-step virtual screening protocol linked with binding free energy and ADMET/pose filtering to identify the putative natural compounds as PD-L1 inhibitors for cancer immunotherapy.

## 2. Methods

### 2.1. Receptor and Ligand Library Collection

The three-dimensional (3D) crystal structure of the human programmed death ligand 1 (PD-L1) co-crystallized with (2~[20],4~[20])-1-[[5-chloranyl-2-[(3-cyanophenyl)methoxy]-4-[[3-(2,3-dihydro-1,4-benzodioxin-6-yl)-2-methyl-phenyl]methoxy]phenyl]methyl]-4-oxidanyl-pyrrolidine-2-carboxylic acid (JQT) inhibitor at 2.20 Å resolution was retrieved as receptor from the RCSB Protein Data Bank (https://www.rcsb.org/, accessed on 10 December 2021; PBD ID: 6R3K) [38]. Additionally, a total of 32,552 natural compounds were downloaded as a ligand library from the Natural Product Atlas database (https://www.npatlas.org/, accessed on 3 December 2021) [39] for multi-step structure-based virtual screening protocol against PD-L1 receptor.

### 2.2. Multi-Step Virtual Screening and Pose Filtration

Multi-step virtual screening of the ligands against PD-L1 was performed using a virtual screening workflow in the Schrödinger suite 2020-4 [40]. Initially, the pre-processing of the PD-L1 protein as receptor was performed using the PRIME tool [41,42] and protein preparation wizard [43] in the Maestro-Schrödinger suite 2020-4 [40]. Briefly, the co-crystallized water molecules were removed from the protein structure, which may obstruct the ligand interaction with the protein, while polar hydrogen atoms were added based on the hybridization of carbon atoms followed by protein structure refinement under default parameters using the Protein preparation wizard. Following, the key residues, viz. Phe^19^, Ile^54^, Tyr^56^, Met^115^, Ile^116^, Ala^121^, Asp^122^, Tyr^123^, Lys^124^, and Arg^125^ of Chain A and Tyr^56^, Gln^66^, Met^115^, Ile^116^, Ser^117^, Ala^121^, and Asp^122^ of chain B, in PD-L1 structure showing interactions with the co-crystalized ligand, i.e., JQT inhibitor, were considered for docking grid generation to perform the multi-step structure-based virtual screening under default parameters using GLIDE v8.9 tool in the Maestro-Schrödinger suite 2020-4 [44]. 

Likewise, 32,552 natural compounds were prepared as ligands under default parameters using LigPrep module tool in the Schrödinger suite 2020-4 [45]. Briefly, in the virtual screening workflow tool, all the ligands were filtered for the ADME (absorption, distribution, metabolism, and excretion) and drug-likeness criteria using the QikProp tool [46]. Following, the filtered ligands were virtually screened via three subsequent steps, including (i) high throughput virtual screening (HTVS), (ii) standard precision (SP) screening, and (iii) extra precision (XP) screening protocol, where only 10% of the top screened ligands from the first step were considered in next successive step to collect the most potent ligands against the PD-L1 protein. Finally, the post-process binding free energy calculations, based on molecular mechanics generalized Born surface area (MM/GBSA) method, were performed on the screened poses under default parameters with OPLS (Optimized Potentials for Liquid Simulations)-3e force field in Prime MM/GBSA module of Maestro-Schrödinger suite 2020-4 [40,42]. The binding free energy calculation was conducted on the screened poses to distinguish the most suitable docked conformations of the natural compounds with the PD-L1 protein for further computational analysis. The equations, used to calculate binding free energy and the associated energy dissociation components for each protein–ligand system, are described in the mathematical Equations (1)–(3).

(1) $$\Delta G_{\mathrm{Bind}}=\Delta G_{\mathrm{Com}}-\left(\Delta G_{\mathrm{Rec}\;}+\;\Delta G_{\mathrm{Lig}}\right)=\Delta H-T\Delta S\;\approx \;\Delta E_{\mathrm{MM}}+\Delta G_{\mathrm{sol}}-T\Delta S\;\;$$

(2) $$\Delta E_{\mathrm{MM}}=\Delta E_{\mathrm{Int}}+\Delta E_{\mathrm{Ele}\;}+\Delta E_{\mathrm{vdW}}\;$$

(3) $$\Delta G_{\mathrm{Sol}}=\Delta G_{\mathrm{Pol}}+\Delta E_{\mathrm{Nonpol}}\;$$

In the above-mentioned equations, Δ*G*_Bind_ and Δ*G*_Com_ denote the binding free energy, or Gibbs free energy, and the total free energy of a docked complex containing protein and ligand, respectively. The sum of the protein and ligand in their free state is denoted by the Δ*G*_Rec_ + Δ*G*_Lig_. The Δ*G*_Bind_ can also be computed from enthalpy (Δ*H*) and entropy (−*T*Δ*S*) for the whole system under consideration using the second law of thermodynamics (Equation (1)). Here, in this study, the entropy contributing to the net Δ*G*_Bind_ was not calculated for the protein–ligand complexes due to the unavailability of expensive computational calculations. In addition, the entropy seems to have a low contribution to the net Δ*G*_Bind_ for similar systems, as reported earlier [47,48,49,50,51]. Therefore, Δ*G*_Bind_ of the protein–ligand complex is designated equivalent to Δ*H* only, which is expressed as the sum of solvation free (Δ*G*_Sol_) and molecular mechanical (Δ*E*_MM_) energy (Equation (2)). Usually, Δ*E*_MM_ constitutes the intermolecular, electrostatic, and van der Waals interactions energies represented as Δ*E*_Int_, Δ*E*_Ele_, and Δ*E*_vdW_, respectively, whereas the Δ*G*_Sol_ constitutes polar and non-polar energies represented as Δ*G*_Pol_ and Δ*E*_Nonpol_, respectively, for the whole system. Hence, the Δ*G*_Bind_ for each docked protein–ligand complex was calculated using Prime MM/GBSA module under default parameters using the Prime MM/GBSA module of Maestro-Schrödinger suite 2020-4, as reported earlier [52,53].

Moreover, the crystal structure of PD-L1 with JQT inhibitor in the protein crystal structure was also docked in the protein pocket under similar conditions and used as a reference complex for the comparative analysis with the docked complexes of PD-L1 with screened natural compounds. The intermolecular interaction analysis of the docked complexes was extracted at 4 Å around the ligand in the binding pocket of the PD-L1 protein under default parameters of the Maestro-Schrödinger suite 2020-4. All 2D and 3D images of both ligand and receptor were rendered using the free academic version of the Maestro v12.6 tool of Schrödinger suite 2020-4 [54].

### 2.3. Molecular Dynamic Simulation

The best-docked poses of PD-L1 with natural compounds were studied for their dynamic stability and intermolecular interaction profiling as a function of 100 ns interval under explicit solvent molecular dynamics (MD) simulation on a Linux environment over HP Z2 Microtower workstation using the free academic version of Desmond v5.6 [55] module in Maestro-Schrödinger suite 2018–4 [56]. Herein, each complex was placed in the center of the orthorhombic grid box (10 Å × 10 Å × 10 Å) solvated with TIP4P (transferable intermolecular potential 4 points) to collect maximum solvation effects of the natural solvent. Additionally, the whole system was neutralized using the counter sodium and chlorine ions while placed at 20 Å around the docked ligand within the binding pocket of the PD-L1 protein. Moreover, 0.15 M salt was added to the system to mimic the physiological conditions for the docked complex using the system building tool. Furthermore, the complete system was subjected to 2000 steps and a 1.0 kcal/mol/Å convergence threshold for the initial minimization using minimization tool. Eventually, the unrestrained 100 ns MD simulation trajectories were generated for each complex under a normal temperature and pressure (NPT) ensemble at 300 K with a 10 ps step size under default parameters with Optimized Potentials for Liquid Simulations (OPLS)-2005 force field, and later produced trajectories were analyzed using the simulation interaction diagram (SID) tool in free academic Desmond v5.6 module with Maestro-Schrödinger suite 2018–4 interface [56].

### 2.4. End-Point Binding Free Energy Calculation

The end-point binding free energy calculations were conducted on the complete 100 ns MD simulation trajectory of each docked complex under the OPLS-3e force field using the Prime MM/GBSA module [40,42], as described earlier under section, ‘Multi-step virtual screening and pose filtration’. Herein, snapshots collected at every 10 ps were treated for the removal of explicit solvent and ions molecules, and computed binding free energy for each complex is depicted as a function of 100 ns and as the mean with standard error.

## 3. Result and Discussion

### 3.1. Virtual Screening and ADMET Analysis

Virtual screening techniques are commonly used in the drug discovery pipelines to identify a ligand with considerable binding affinity with a receptor from the large compound databases and evaluate the ligand binding energy in terms of scoring functions [57,58,59]. However, accuracy remains a major limitation contributed by the least conformational sampling of ligand and reliability of approximate scoring functions implemented in virtual screening applications. This may result in the collection of false-positive and false-negative hits, which then require rigorous assessment before further computational analysis [59,60,61]. In this context, the refinement of the generated poses using binding free energy calculations have been demonstrated as an ideal method to distinguish the positive hits and ranking of cognate ligands identified using virtual screening applications [62,63,64]. Notably, MM/GBSA method has been reported to accurately assess the binding free energy between protein and small-molecule ligands [47,65,66].

**Figure 1:**
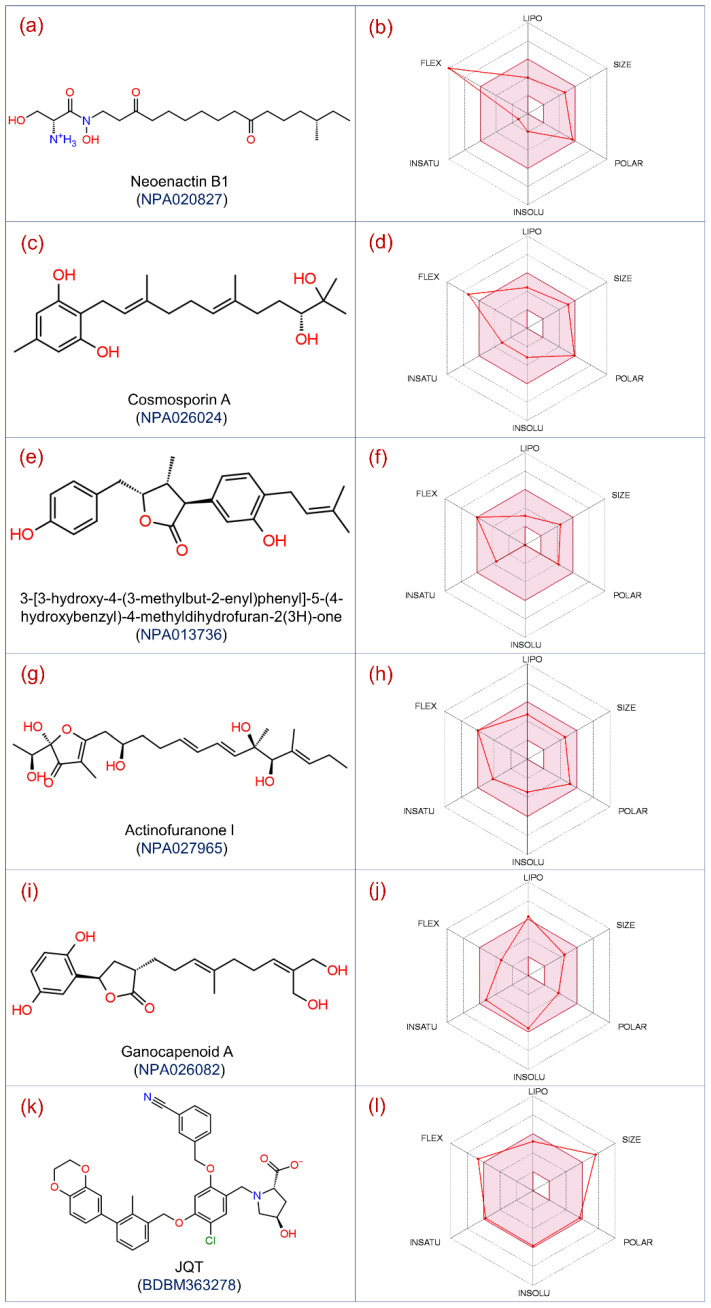
2D structures and ADMET of selected natural compounds, i.e., (**a**,**b**) Neoenactin B1, (**c**,**d**) Cosmosporin A, (**e**,**f**) 3-[3-hydroxy-4-(3-methylbut-2-enyl)phenyl]-5-(4-hydroxybenzyl)-4-methyldihydrofuran-2(3H)-one Cosmosporin A, (**g**,**h**) Actinofuranone I, (**i**,**j**) Ganocapenoid A, and (**k**,**l**) JQT inhibitor as the reference ligand, selected for the computational analysis against the PD-L1 protein.

In this study, a total of 173,403 conformations were generated for the 32,552 natural compounds using the Ligprep tool and then processed through drug-likeness filters, followed by three levels of structure-based virtual screening, i.e., HTVS, SP, and XP protocols. Furthermore, the selected poses were evaluated for the selection of the most ideal docked conformation of natural compounds, with PD-L1 using post-docking MM/GBSA method, resulting in an assortment of total 17 natural compounds against the PD-L1 protein. The collected natural compounds were noted for considerable ADMET properties (Supporting Information Table S1), and substantial docking scores (>−10 kcal/mol) and binding free energy (>−40 kcal/mol) in the targeted binding pocket of the PD-L1 protein (Table 1). Thus, based on the highest binding free energy values, the top five docked poses of PD-L1 with natural compounds, i.e., Neoenactin B1, Actinofuranone I, Cosmosporin A, Ganocapenoid A, and 3-[3-hydroxy-4-(3-methylbut-2-enyl)phenyl]-5-(4-hydroxybenzyl)-4-methyldihydrofuran-2(3H)-one, were marked with high potency and selected for further intermolecular interaction analysis (Figure 1). Of note, Neoenactin B1, isolated as the antifungal compound from *Streptomyces olivoreticuli* [67], and Actinofuranone I, isolated from *Streptomyces gramineus*, were reported for anti-inflammatory properties [68]. Likewise, isolation of Cosmosporin A, Ganocapenoid A, and 3-[3-hydroxy-4-(3-methylbut-2-enyl)phenyl]-5-(4-hydroxybenzyl)-4-methyldihydrofuran-2(3H)-one, were reported from the fungi *Pseudocosmospora* [69], *Ganoderma capense* [70], and *Aspergillus terreus* [71], respectively, and these compounds are not reported for considerable biological activity.

### 3.2. Docking Pose Validation and Interaction Analysis

**Figure 2:**
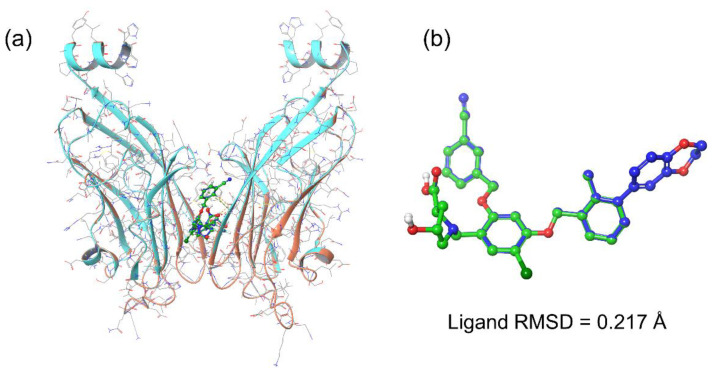
Alignment of crystal structure and docked poses of the JQT inhibitor in the binding pocket of PD-L1 protein. Herein, (**a**) orange (crystal structure) and light blue (docked structure) colors represent the protein while (**b**) dark blue (co-crystalized) and green (docked ligand) depict the 3D structures of the JQT inhibitor.

Initially, the co-crystallized ligand, i.e., JQT inhibitor, was docked in the targeted binding pocket of the PD-L1 protein followed by the selection of an ideal conformation based on binding free energy (−63.98 kcal/mol) (Table 1). To validate the selected pose of the reference complex, docked conformation of the JQT inhibitor was aligned to co-crystallized conformation in the PD-L1 structure (PBD ID: 6R3K) using the Structure superimpose tool in the Maestro-Schrödinger suite 2020.4 (Figure 2). Interestingly, docked conformation showed absolute matching with 0.217 Å root mean square deviation (RMSD) aligned on the native conformation of the JQT inhibitor in the crystal structure of the PD-L1 protein. These results support the considered binding pocket in the identification of putative natural compounds as inhibitors of PD-L1 protein; hence, the respective docked poses were considered for further computational analysis.

The molecular interaction analysis for the protein–ligand docked complex is employed to determine the effectiveness of the compound against a target in the structure-based drug discovery approaches [72]. Thus, molecular docked poses of the selected five natural compounds, i.e., Neoenactin B1, Actinofuranone I, Cosmosporin A, Ganocapenoid A, and 3-[3-hydroxy-4-(3-methylbut-2-enyl)phenyl]-5-(4-hydroxybenzyl)-4-methyldihydrofuran-2(3H)-one, as putative inhibitors and the JQT inhibitor as the reference ligand were studied for the residual interactions at 4 Å radius around the docked ligand in the binding pocket of the PD-L1 protein (Table 2).

**Figure 3:**
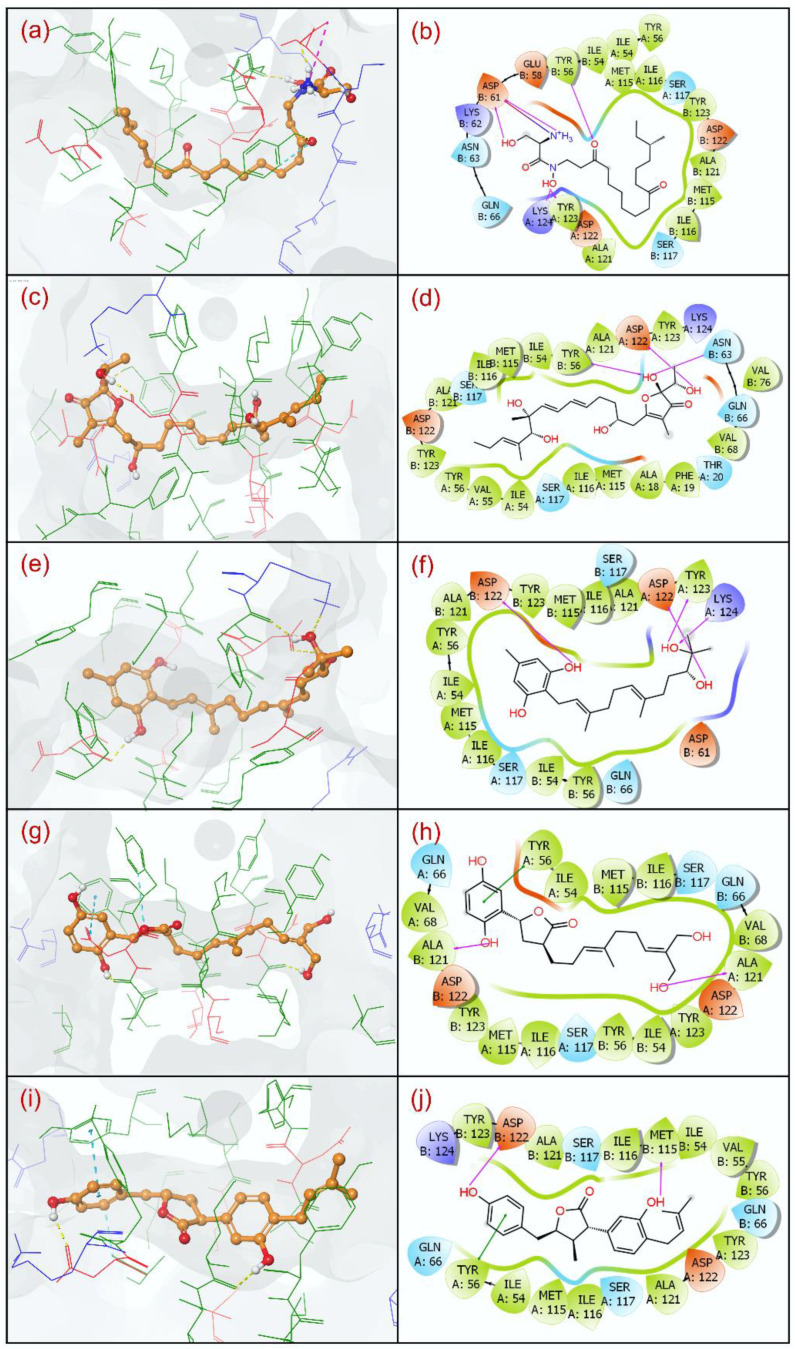
3D and 2D poses of the selected natural compounds, i.e., (**a**,**b**) Neoenactin B1, (**c**,**d**) Actinofuranone I, (**e**,**f**) Cosmosporin A, (**g**,**h**) Ganocapenoid A, and (**i**,**j**) PD-L1-3-[3-hydroxy-4-(3-methylbut-2-enyl)phenyl]-5-(4-hydroxybenzyl)-4-methyldihydrofuran-2(3H)-one, collected at 4 Å space around the ligand within in the docked site of the PDL-1 protein. In 2D interaction maps, pink arrow (H-bond), green line (π-π stacking, red-violet (salt bridge), red (negative), violet (positive), green (hydrophobic), and blue (polar) color residues exhibits the interactions in the respective docked complexes.

The analysis of docked PD-L1-Neoenactin B1 complex shows the formation of five hydrogen bonds (H-bonds) with A:Tyr^123^, A:Lys^124^, B:Tyr^56^, and B:Asp^61^(2) residues; PD-L1-Actinofuranone I complex also displays establishment of three H-bonds with A:Asp^122^, B:Tyr^56^, and B:Asn^63^ residues; PD-L1-Cosmosporin A complex exhibits four H-bonds formation with A:Asp^122^, A:Tyr^123^, A:Lys^124^, and B:Asp^122^ residues; PD-L1-Ganocapenoid A complex depicts two H-bonds interaction with A:Ala^121^ and B:Ala^121^ residues; and PD-L1-3-[3-hydroxy-4-(3-methylbut-2-enyl)phenyl]-5-(4-hydroxybenzyl)-4-methyldihydrofuran-2(3H)-one complex shows formation of two H-bonds with B:Ala^122^ and B:Met^115^ residues (Table 2, Figure 3).

However, no H-bond formation was observed in the reference complex, i.e., PD-L1-JQT inhibitor (Table 2, Supporting Information Figure S1). Additionally, only PD-L1-Neoenactin B1 complex was noted for salt-bridge formation with B:Asp^61^ residue in comparison to PD-L1-JQT inhibitor complex (A:Lys^124^). Moreover, PD-L1-Ganocapenoid A and PD-L1-3-[3-hydroxy-4-(3-methylbut-2-enyl)phenyl]-5-(4-hydroxybenzyl)-4-methyldihydrofuran-2(3 H)-one complexes exhibited π-π stacking interactions at A:Tyr^56^ residue in comparison to PD-L1-JQT inhibitor complex, which showed both π-π stacking (B:Tyr^56^) and π-cation stacking interactions (A:Lys^124^). Additionally, the docked natural compounds and the JQT inhibitor in the binding pocket of PD-L1 were also observed for other residual interactions, involving hydrophobic, polar, positive, and negative interactions (Table 2, Figure 3, Supporting Information Figure S1). Notably, interacting residues with the natural compounds were also noted in the interaction map of the reference complex (residues are highlighted as ‘bold text’ in Table 1), indicating the natural compounds have relatively occupied the same binding pocket as the reference ligand. Thereof, based on the binding energy profiles (docking scores and binding free energy) in association with the observed intermolecular interactions of the docked natural compounds against PD-L1-JQT inhibitor complex, the docked natural compounds in the binding pocket of PD-L1 are suggested to have substantial stability by comparison to the JQT inhibitor, and may contribute to inhibition of the PD-1/PD-L1 signaling pathway, as reported for the JQT inhibitor [38].

### 3.3. Molecular Dynamic Simulation Analysis

To predict the dynamic stability and intermolecular interactions as a function of 100 ns, classical MD simulation was performed on each docked PD-L1-natural compound, viz. Neoenactin B1, Actinofuranone I, Cosmosporin A, Ganocapenoid A, and 3-[3-hydroxy-4-(3-methylbut-2-enyl)phenyl]-5-(4-hydroxybenzyl)-4-methyldihydrofuran-2(3H)-one complex, and analyzed in comparison to the apo-PD-L1 protein and reference docked complex, i.e., PD-L1-JQT inhibitor complex, MD simulation trajectories.

**Figure 4:**
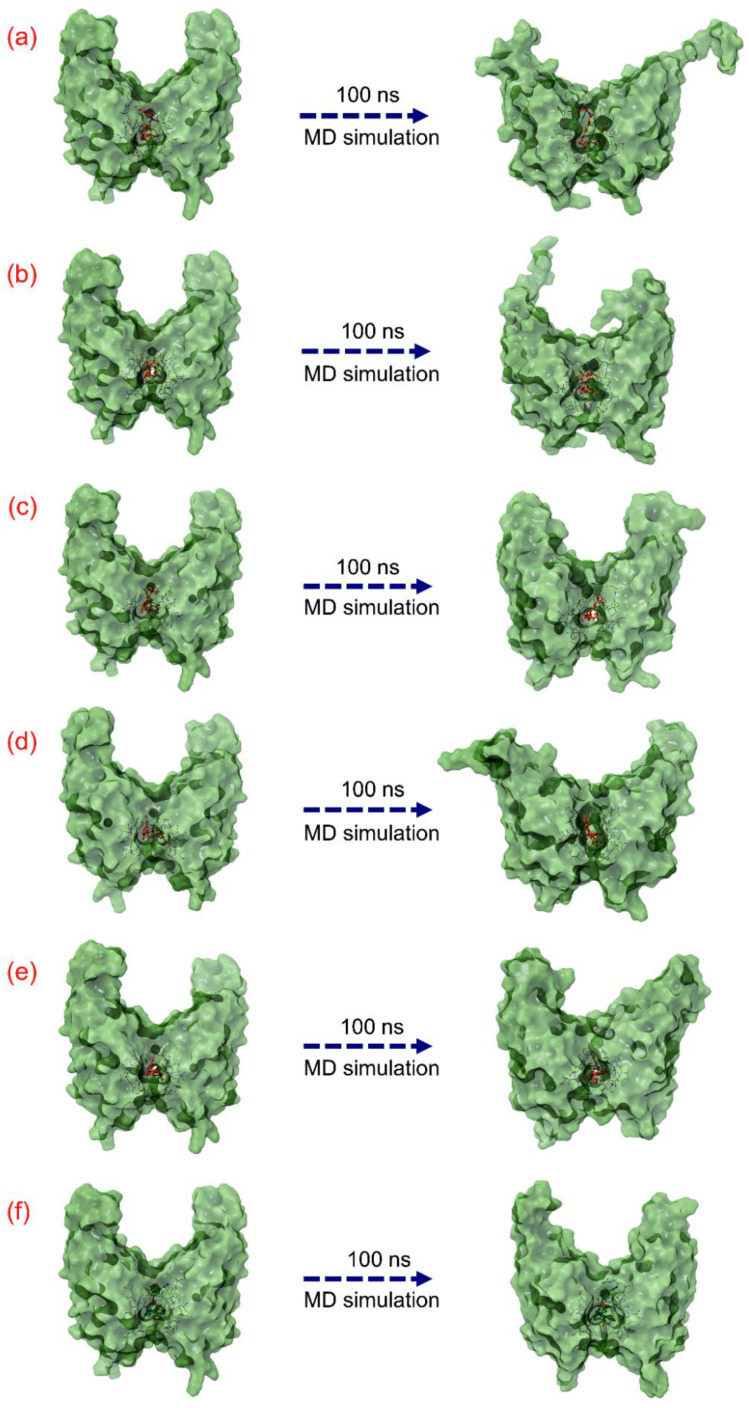
Representation of the 3D surface conformational changes in the last poses, i.e., (**a**) PD-L1-Neoenactin B1, (**b**) PD-L1-Actinofuranone I, (**c**) PD-L1-Cosmosporin A, (**d**) PD-L1-Ganocapenoid A, (**e**) PD-L1-3-[3-hydroxy-4-(3-methylbut-2-enyl)phenyl]-5-(4-hydroxybenzyl)-4-methyldihydrofuran-2(3H)-one, and (**f**) JPD-L1-JQT inhibitor, extracted from the 100 ns MD simulation trajectories in comparison to the respective docked poses.

Initially, the last poses of the 100 ns MD simulations were recovered and compared with respective docked poses for molecular contacts analysis to assure the residence of the docked natural compounds as ligand in the considered binding pocket of the PD-L1 protein via constant or similar number of residual interactions and studied for conformational changes in both proteins and ligands (Table 3, Figure 4). Notably, all the last poses of the docked natural compounds with PD-L1 receptor after 100 ns MD simulation were observed for consistent residual interactions in comparison to the respective docked poses, supporting the stability of all the docked natural compounds in the binding pocket of PD-L1 receptor in comparison to the reference complex (Table 3). Moreover, 3D surface analysis of the last poses from the 100 ns MD simulation reveals substantial conformational changes in the protein structure docked with natural compounds against the PD-L1 protein docked with the JQT inhibitor, suggesting the potential of docked natural compounds to significantly disturb the native conformational of the PD-L1 protein (Figure 4). Furthermore, the MD simulation trajectories of the respective docked complexes were statistically analyzed in terms of root mean square deviation (RMSD), root mean square fluctuation (RMSF), and protein–ligand interaction fraction mapping to understand the dynamic stability of the docked complexes as a function of 100 ns MD simulation interval. 

### 3.4. RMSD and RMSF Analysis

**Figure 5:**
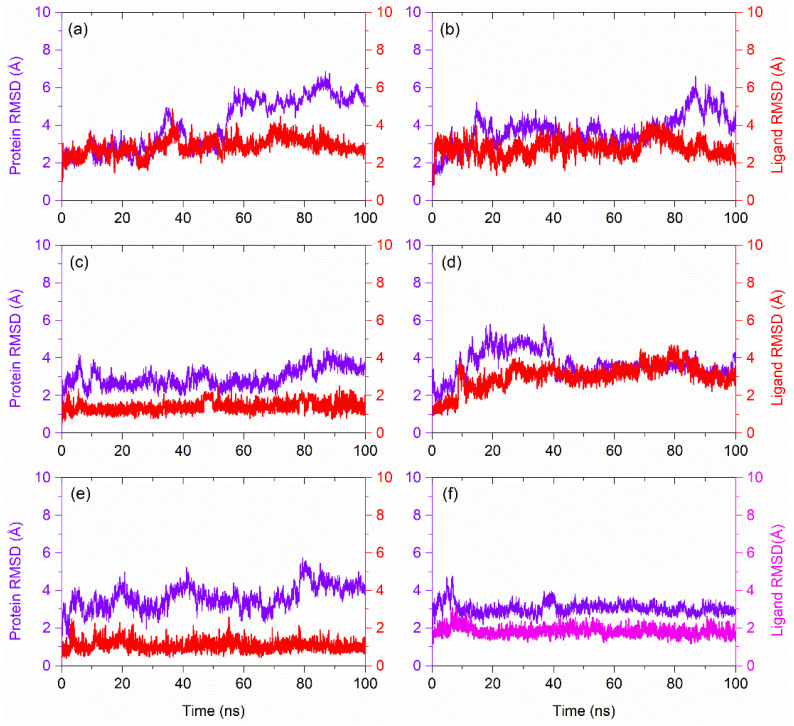
RMSD plots for the PD-L1-natural compounds, i.e., (**a**) Neoenactin B1, (**b**) Actinofuranone I, (**c**) Cosmosporin A, (**d**) Ganocapenoid A, (**e**) 3-[3-hydroxy-4-(3-methylbut-2-enyl)phenyl]-5-(4-hydroxybenzyl)-4-methyldihydrofuran-2(3H)-one, and (**f**) PD-L1-JQT inhibitor complexes as function of 100 ns simulation interval. Herein, protein RMSD values were extracted in terms of alpha carbon atoms while ligand RMSD values were computed as the protein-fit ligand for all the docked complexes from their respective 100 ns MD simulation trajectories.

Initially, RMSD values were computed from the respective docked poses of PD-L1-natural compounds as a function of 100 ns simulation interval and analyzed in comparison to the RMSD values of apo-protein and PD-L1-JQT inhibitor complex (Figure 5, Supporting Information Figure S2). In all the docked complexes of PD-L1 with natural compounds, substantial deviations (>3.5 Å) were observed in the protein throughout 100 ns MD simulation, except in PD-L1-Ganocapenoid A complex, where equilibrium in the protein RMSD (~4 Å) was noted after 40 ns until the end of the 100 ns MD simulation, while PD-L1 protein exhibited higher deviations (>4.8 Å) in PD-L1-Neoenactin B1 and PD-L1-Actinofuranone I docked complexes during the simulation interval. However, PD-L1 docked with the JQT inhibitor showed high deviation (~4 Å) within the first 10 ns interval followed by a state of global minima (~3 Å) until the end of simulation, while apo-PD-L1 receptor was also noted for consistent deviations (<4.2 Å) on several occasions without a state of equilibrium during the MD simulation interval. These observations suggested that, unlike the JQT inhibitors, docked natural compounds, particularly Neoenactin B1 and Actinofuranone I, may promote the strong global conformational changes in the PD-L1 protein. These observations were further supported by the calculated RMSF values (>2.5 Å) for the docked PD-L1 with Neoenactin B1 and Actinofuranone I natural compounds in comparison to apo-protein (<2.5 Å) and protein docked with the JQT inhibitor (<2.5 Å) (Supporting Information Figures S2 and S3).

Likewise, protein-fit ligand RMSD analysis indicated substantial global minima (<3 Å) for all the docked natural compounds throughout the 100 ns MD simulation interval, except for the Cosmosporin A and 3-[3-hydroxy-4-(3-methylbut-2-enyl)phenyl]-5-(4-hydroxybenzyl)-4-methyldihydrofuran-2(3H)-one compounds, which showed <2 Å RMSD as observed for the JQT inhibitor (<2 Å) (Figure 5). Moreover, computed ligand RMSF also reveals the acceptable values (<3 Å) for all the docked natural compounds with PD-L1 against the JQT inhibitor (Supporting Information Figure S4), suggesting the substantial stability of the docked ligand with the protein. Collectively, RMSD and RMSF analysis of the docked complexes suggested the considerable stability of the docked ligands with the protein, while docked natural compounds were noted to induce substantial conformational changes in the PD-L1 protein structure that may results in the inhibition of PD-L1 protein with its receptor in the PD-1/PL-1 pathway.

### 3.5. Protein–Ligand Interaction Mapping

**Figure 6:**
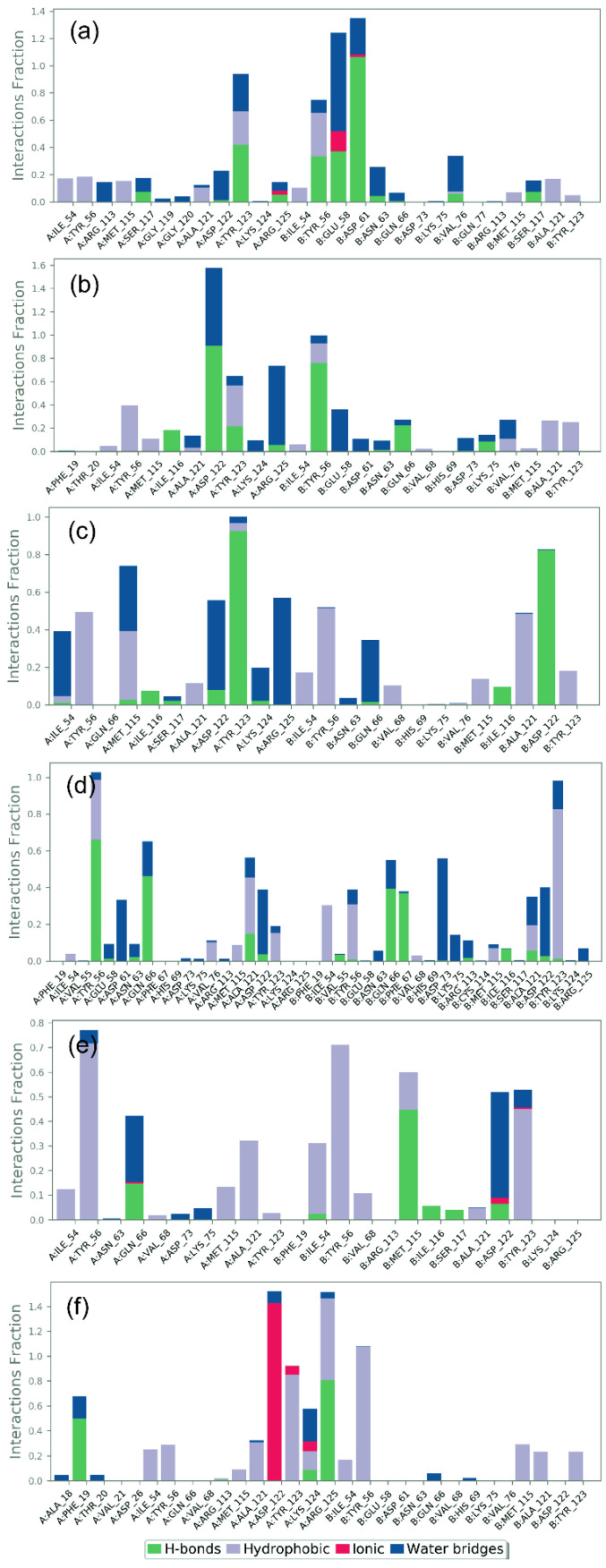
Protein–ligand interactions mapping PD-L1 and selected natural compounds, i.e., (**a**) Neoenactin B1, (**b**) Actinofuranone I, (**c**) Cosmosporin A, (**d**) Ganocapenoid A, (**e**) 3-[3-hydroxy-4-(3-methylbut-2-enyl)phenyl]-5-(4-hydroxybenzyl)-4-methyldihydrofuran-2(3H)-one, and (**f**) JQT inhibitor, fit on protein were extracted from 100 ns MD simulation trajectories of respective docked complexes.

To further access the stability of the docked complexes in terms of intermolecular interactions as a function of simulation interval, protein–ligand contact maps, including H-bonding, hydrophobic interactions, ionic interactions, and water bridge formation, were extracted from the respective 100 ns MD simulation trajectories (Figure 6). Notably, all the docked natural compounds showed considerable molecular contacts with the active residues in the binding pocket of the PD-L1 protein during the simulation interval in comparison to the reference complex, i.e., PD-L1-JQT inhibitor; the interacting residues were also noted in the initially docked poses (Table 2).

In the PDL1-Neoenactin B1 complex (Figure 5a), B:Asp^61^ (which formed two H-bonds in docked complex) exhibited substantial H-bond formation for ~100% of the interaction fraction, while B:Tyr^56^ (which formed single H-bond in docked complex) and A:Tyr^123^ (noted for both H-bond and hydrophobic interactions in docked complex) were noted for hydrophobic interactions with docked ligands for ~20% of the total interaction fraction during the simulation interval. Additionally, B:Glu^58^ (which depicted negative residual interaction in docked complex) showed ~50% and ~15% of total interaction fractions in water bridge formation and ionic bond formations, respectively, during the 100 ns simulation interval.

Likewise, in the PDL1-Actinofuranone I complex (Figure 5b), A:Asp^122^ (which showed both H-bond and negative residual interaction in docked complex) residue contributed in ~90% of the interaction fraction for H-bond formation, in addition to water bridge formation (~60% interaction fraction) during the 100 ns simulation interval. Moreover, A:Tyr^56^ (which exhibited both H-bond and hydrophobic interaction in docked complex) and A:Tyr^123^ (which displayed hydrophobic interaction in docked complex) showed ~30% of the interaction fraction in hydrophobic interactions, A:Arg^125^ (observed for intermolecular interaction during MD simulation only) exhibited water bridge formation (~50% interaction fraction), and B:Tyr^56^ (which showed both H-bond and hydrophobic interaction in docked complex) formed H-bond (~70% of the interaction fraction) during total simulation interval.

Additionally, protein–ligand contact analysis of the PDL1-Cosmosporin A complex showed substantial contribution of A:Tyr^56^, B:Tyr^56^, and B:Ala^121^ (all three residues also showed hydrophobic interactions in docked complex) in hydrophobic interaction (~50% of the interaction fraction); A:Tyr^123^ (which displayed H-bond and hydrophobic interactions in docked complex) and B:Asp^122^ (which displayed H-bond formation in docked pose) noted ~90% and ~85% of interaction fraction in H-bond formation, respectively; and A:Arg^125^ (observed for interaction during MD simulation only) presented water bridge formation (~55% of the interaction fraction) during the 100 ns MD simulation interval (Figure 5c).

Furthermore, analysis of the PD-L1-Ganocapenoid A complex showed substantial contribution of A:Tyr^56^ (noted for π-π stacking interaction in docked complex) and A:Gln^66^ (distinguished for polar interaction in docked complex) in H-bond formation for ~65% and ~55% interaction fraction, respectively; B:Asp^73^ (detected for interaction during MD simulation only) exhibits water bridge formation (~55% interaction fraction), and B:Tyr^123^ (which displayed hydrophobic interaction in docked pose) was noted for hydrophobic interactions (~85% interaction fraction) during the total simulation interval (Figure 5d).

Whilst analysis of protein–ligand mapping of the PD-L1-3-[3-hydroxy-4-(3-methylbut-2-enyl)phenyl]-5-(4-hydroxybenzyl)-4-methyldihydrofuran-2(3H)-one complex showed the significant contribution of A:Tyr^56^ (which showed both hydrophobic and π-π stacking interaction in docked complex) and B:Tyr^56^ (which presented hydrophobic interaction in docked complex) in hydrophobic interaction for ~70% interaction fraction, B:Tyr^123^ (which displayed hydrophobic interaction in docked pose) also demonstrated hydrophobic interaction (~45% interaction fraction); B:Met^115^ (which displayed H-bond formation in docked pose) contributed in H-bond formation (~45% interaction fraction), and B:Asp^122^ (which showed both H-bond and negative residual interaction in docked complex) revealed water bridge formation (~40% interaction fraction) during the 100 ns MD simulation interval (Figure 5e).

The protein–ligand mapping of the PD-L1 protein with its native ligand, i.e., the JQT inhibitor, as reference complex substantially demonstrated ionic interaction (~45% interaction fraction) via A:Asp^122^ (which displayed negative residual interaction in docked complex), hydrophobic interaction (~100% interaction fraction) via B:Tyr^56^ (which displayed hydrophobic interaction in docked pose), hydrophobic interaction (~80% interaction fraction) via A:Tyr^123^ (which exhibited hydrophobic interaction in docked complex), H-bond formation (80% interaction fraction) via A:Arg^125^ (which showed positive residual interaction in docked pose), and water bridge formation (20% interaction fraction) via A:Lys^124^ (which showed π-cation stacking, salt bridge, and positive residual interaction in docked pose) residues as a function of the 100 ns simulation interval (Figure 5f).

Additionally, molecular contact formation between the receptor and docked natural compounds were also logged at ~30% of the total simulation interval, indicating substantial contribution of H-bonding and hydrophobic interactions in comparison to the reference complex (Supporting Information Figure S5). Altogether, collected interaction profiles as a function of simulation interval indicate a substantial contribution of H-bonds and hydrophobic interactions in the dynamic stability of PD-L1-natural compounds complexes during the 100 ns MD simulations. Hence, based on 100 ns MD simulation trajectories analysis, the selected docked complexes can be arranged in order of stability, i.e., PDL1-Neoenactin B1, PDL1-Cosmosporin A, PDL1-Actinofuranone I, PDL1-Ganocapenoid A, and PDL1-3-[3-hydroxy-4-(3-methylbut-2-enyl)phenyl]-5-(4 hydroxybenzyl)methyldihydrofuran an-2(3H)-one, in comparison to the reference complex, viz. PD-L1-JQT inhibitor.

### 3.6. End-Point Binding Free Energy Analysis

**Figure 7:**
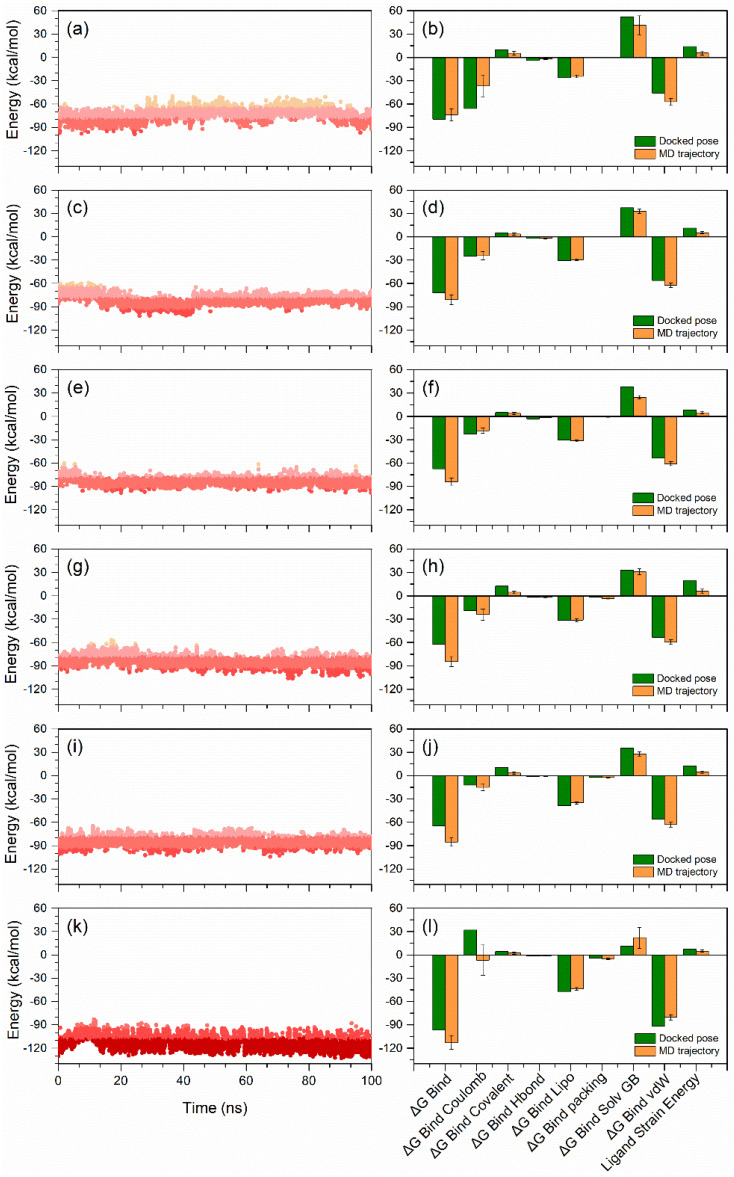
Binding free energy and individual dissociation energy components calculation performed for PD-L1 and selected natural compounds, i.e., (**a**,**b**) Neoenactin B1 (**c**,**d**) Actinofuranone I (**e**,**f**) Cosmosporin A, (**g**,**h**) Ganocapenoid A (**i**,**j**) 3-[3-hydroxy-4-(3-methylbut-2-enyl)phenyl]-5-(4-hydroxybenzyl)-4-methyldihydrofuran-2(3H)-one, and (**k**,**l**) JQT inhibitor before and after 100 ns MD simulation.

In addition, to understand the macromolecular system at atomic level, the application of MD simulation is also helpful to decipher the hidden or undetected states of system under consideration [73,74,75]. Thus, as end-point free energy methods [76,77,78], MD simulations in combination with binding free energy methods have been comprehensively used in structure-based drug design to determine the thermodynamic properties of the macromolecular system, including stability, affinity, and free energy decomposition analysis [75]. Therefore, the most well-known end-point free energy MM/GBSA method, which provides an ideal balance between accuracy and computational efficiency, was utilized on the complete 100 ns MD simulation trajectories to calculate the end-point binding free energy for each PD-L1-ligand complex (Supporting Information Table S2, Figure 7).

Initially, calculated binding free energy for the complete 100 ns simulation trajectories of the docked PD-L1 with natural compounds were analyzed in comparison to the reference complex. Although PD-L1-natural compounds showed prominent binding free energy between −60 to −90 kcal/mol against PD-L1-JQT inhibitor (−90 to −130 kcal/mol), some conformations also exhibited higher energy >−90 kcal/mol during the simulation interval. As expected, these conformations represent the most stable poses of the ligand compounds with the PD-L1 protein (Figure 7). These observations suggested that docked natural compounds may exhibit higher binding affinities than the predicted binding affinities using MM/GBSA method.

Furthermore, average binding free energy values for each simulated complex were computed along with energy decomposition components to assess the favorable and unfavorable energy terms to the net biding free energy of the system and compared with the respective energy terms computed on docked poses (Figure 7). Interestingly, all the PD-L1-natural compound complexes showed significant increment in the binding free energy (>−80 kcal/mol) in comparison to the respective docked poses (>−60 kcal/mol), except PD-L1-Neoenactin B1 complex (−73.55 ± 7.62 kcal/mol). Similarly, PD-L1-JQT inhibitor also showed considerable hike in the binding free energy (−112.91 ± 8.33 kcal/mol) after the MD simulation against docked pose (−96.7 kcal/mol). Among the PD-L1 natural compounds, PD-L1-3-[3-hydroxy-4-(3-methylbut-2-enyl)phenyl]-5-(4hydroxybenzyl)methyldihydrofuran-2(3H)-one docked complex (−87.21 ± 4.11 kcal/mol) and PD-L1-Neoenactin B1 complex (−73.55 ± 7.62 kcal/mol) were marked for the highest and lowest end-point binding free energy, respectively, after the 100 ns MD simulation (Figure 7), whereas the end-point binding free energy of the docked complex PD-L1-Actinofuranone I was −81.07 ± 6.41 kcal/mol, followed by −83.96 ± 4.51 kcal/mol for PD-L1-Cosmosporin A, and −84.56 ± 6.36 kcal/mol for PD-L1-Ganocapenoid A (Figure 7).

Furthermore, the computation of dissociation energy components for each complex before and after MD simulation revealed the favorable contribution of Δ*G*_Bind Lipo_ (Lipophilic) and Δ*G*_Bind vdW_ (Van der Waals interaction) energies to the net stability of docked complexes, whereas Δ*G*_Bind Solv GB_ (Generalized Born electrostatic solvation energy) substantially contributed to the instability of the respective docked complexes (Figure 7). Notably, after the 100 ns MD simulation, a decrement in the Δ*G*_Bind Solv GB_ was noted and no substantial difference was observed in the Δ*G*_Bind Lipo_ energy terms. Similar energy dissociation components were previously noted to contribute to the stability of the docked complexes of the PD-L1 protein [79,80]. Furthermore, reduction in net ligand strain energy was also noted following 100 ns MD simulation in each complex in comparison to the respective docked poses, suggesting the favorable contribution to the protein–ligand complex stability after 100 ns MD simulation (Figure 7). Hence, from the comparative binding free energy analysis of docked poses and MD simulation trajectories values, natural compounds are endorsed as potential hit candidates in comparison to the reference compounds, i.e., JQT inhibitor, for the development of PD-L1 inhibitors for cancer immunotherapy.

## 4. Conclusions

Programmed death ligand-1 (PD-L1) is a potential target for the suppression of cancer progression. The development of anti-cancer compounds using natural compounds by inhibiting the PD-L1 protein can be a turning point in the field of cancer immunotherapy. In this study, five natural products, i.e., Neoenactin B1, Actinofuranone I, Cosmosporin A, Ganocapenoid A, and 3-[3-hydroxy-4-(3-methylbut-2-enyl)phenyl]-5-(4hydroxybenzyl)methyldihydrofuran-2(3H)-one, are identified as potential candidates for the PD-L1 protein inhibition, with substantial drug-likeness, docking energy (>−10 kcal/mol), and MM/GBSA binding free energy (>−60 kcal/mol). In addition, the intermolecular interaction profiling of the docked poses and 100 ns molecular dynamic simulation trajectory analysis promotes the substantial contribution of H-bonding and hydrophobic interactions in the stability of docked natural compounds with the PD-L1 protein. Furthermore, calculated net binding free energy on each simulation trajectory supports the stability of the docked complexes in comparison to the reference complex and advocates the selected natural compounds as potent candidates for the development of PD-L1 inhibitors. Overall, the computational investigation of the natural compounds as PD-L1 inhibitors provides a positive endorsement for the selected natural compounds in the eventual designing and development of an effective small molecule-based anti-PD-L1 agent, which may provide PD-L1 inhibition at low concentration, to disturb the PD-1/PD-L1 signaling pathway for the cancer immunotherapy.

## Figures and Tables

**Table 1:** Names and characteristics of the small molecules collected by structure based virtual screening process against the PD-L1 receptor from the NP-Atlas database.

S. No.	Title	Compound	Mol. Formula	Mol. wt.	Origin	Docking Score (kcal/mol)	Δ*G*_Bind_ (kcal/mol)
1	NPA020827	Neoenactin B1	C_20_H_38_N_2_O_5_	386.531	*Streptomyces olivoreticuli subsp. Neoenacticus*	−10.36	−79.63
2	NPA027965	Actinofuranone I	C_23_H_36_O_7_	424.533	*Streptomyces gramineus*	−10.92	−71.44
3	NPA026024	Cosmosporin A	C_22_H_34_O_4_	362.508	*Pseudocosmospora sp. Bm-1-1*	−10.28	−67.43
4	NPA026082	Ganocapenoid A	C_21_H_28_O_6_	376.449	*Ganoderma capense*	−10.54	−66.92
5	NPA013736	3-[3-hydroxy-4-(3-methylbut-2-enyl)phenyl]-5-(4-hydroxybenzyl)-4-methyldihydrofuran-2(3H)-one	C_23_H_26_O_4_	366.456	*Aspergillus terreus*	−10.49	−64.78
6	NPA030364	4-carbglyceryl-3,3′-dihydroxy-5,5′-dimethyldiphenyl ether	C_18_H_20_O_7_	348.352	*Aspergillus versicolor SCSIO 41502*	−10.45	−60.21
7	NPA004673	Not named	C_19_H_16_O_3_	292.334	*Burkholderia pseudomallei*	−10.50	−57.08
8	NPA020009	Sterin A	C_16_H_20_O_6_	308.33	*Stereum hirsutum*	−11.39	−55.39
9	NPA027779	Decarboxyunguidepside A	C_19_H_20_O_5_	328.364	*Aspergillus unguis*	−10.39	−54.51
10	NPA025743	Premacrophorintriol-I	C_22_H_34_O_5_	378.508	*Trichoderma sp. 1212-03*	−10.34	−54.42
11	NPA002619	4′′-Deoxy-5′-Desmethyl-Terphenyllin	C_19_H_16_O_4_	308.333	*Aspergillus sp. YXf3*	−10.56	−54.41
12	NPA018153	Linieodolide A	C_17_H_30_O_6_	330.42	*Bacillus sp. 09ID194*	−10.30	−53.94
13	NPA017629	5′-O-desmethylterphenyllin	C_19_H_16_O_5_	324.332	*Aspergillus sp. YXf3*	−10.62	−53.87
14	NPA011065	Nocarbenzoxazole E	C_16_H_14_N_2_O_5_	314.297	*Nocardiopsis lucentensis DSM 44048*	−10.73	−53.37
15	NPA022801	Floricolin Q	C_18_H_14_O_5_	310.306	*Floricola striata*	−10.81	−52.11
16	NPA015571	Cylindrocarpol	C_23_H_34_O_5_	390.519	*Acremonium sp.*	−11.86	−49.25
17	NPA014938	Baciphelacin	C_22_H_34_N_2_O_6_	422.52	*Bacillus thiaminolyticus IFO 3967/B-1-7*	−10.89	−40.41
18	JQT inhibitor	BDBM363278	C_36_H_33_ClN_2_O_7_	641.1	*Synthetic*	−9.824	−63.98

**Table 2:** Intermolecular interaction profiles for the docked natural compounds conformation with active residues in the binding pocket of the PD-L1 protein.

S. No.	Complex	H-bond	Hydrophobic	Polar	π-π/*π-Cation	Salt Bridge	Positive	Negative
**1**	PD-L1-Neoenactin B1	**A:Tyr^123^,** **A:Lys^124^, B:Tyr^56^, B:Asp^61^(2)**	**A:Ile^54^, A:Tyr^56^, A:Met^115^, A:Ile^116^, A:Ala^121^, A:Tyr^123^, B:Ile^54^, B:Tyr^56^, B:Met^115^, B:Ile^116^, B:Ala^121^, B:Tyr^123^**	**A:Ser^117^, B:Asn^63^, B:Gln^66^, B:Ser^117^**	-	**B:Asp^61^**	**A:Lys^124^,** B:Lys^62^	**A:Asp^122^,** B:Glu^58^, **B:Asp^61^, B:Asp^122^**
**2**	PD-L1-Actinofuranone I	**A:Asp^122^, B:Tyr^56^,** B:Asn^63^	**A:Ala^18^, A:Phe^19^, A:Ile^54^, A:Val^55^, A:Tyr^56^, A:Met^115^, A:Ile^116^, A:Ala^121^, A:Tyr^123^, B:Ile^54^, B:Tyr^56^, B:Val^68^,** B:Val^76^, **B:Met^115^, B:Ile^116^, B:Ala^121^, B:Tyr^123^**	**A:Thr^20^, A:Gln^66^, A:Ser^117^, B:Asn^63^,** **B:Ser^117^**	-	-	**A:Lys^124^**	**A:Asp^122^, B:Asp^122^**
**3**	PD-L1-Cosmosporin A	**A:Asp^122^,** **A:Tyr^123^, A:Lys^124^,** **B:Asp^122^**	**A:Ile^54^, A:Tyr^56^, A:Met^115^,** **A:Ile^116^, A:Ala^121^, A:Tyr^123^,** **B:Ile^54^, B:Tyr^56^, B:Met^115^, B:Ile^116^, B:Ala^121^, B:Tyr^123^**	**A:Ser^117^, B:Gln^66^** **B:Ser^117^**	-	-	**A:Lys^124^**	**A:Asp^122^, B:Asp^61^** **B:Asp^122^**
**4**	PD-L1-Ganocapenoid A	**A:Ala^121^,** **B:Ala^121^**	**A:Ile^54^, A:Tyr^56^,** A:Val^68^, **A:Met^115^, A:Ile^116^, A:Ala^121^,** A:Tyr^123^, **B:Ile^54^, B:Tyr^56^, B:Val^68^, B:Met^115^, B:Ile^116^, B:Ala^121^, B:Tyr^123^**	**A:Gln^66^, A:Ser^117^,** **B:Gln^66^, B:Ser^117^**	**A:Tyr^56^**	-	-	**A:Asp^122^, B:Asp^122^**
**5**	PD-L1-3-[3-hydroxy-4-(3-methylbut-2-enyl)phenyl]-5-(4-hydroxybenzyl)-4-methyldihydrofuran-2(3H)-one	B:Ala^122^, **B:Met^115^**	**A:Ile^54^, A:Tyr^56^, A:Met^115^, A:Ile^116^,** **A:Ala^121^, A:Tyr^123^, B:Ile^54^,** B:Val^55^, **B:Tyr^56^, B:Met^115^, B:Ile^116^, B:Ala^121^, B:Tyr^123^**	**A:Gln^66^, A:Ser^117^, B:Gln^66^, B:Ser^117^**	**A:Tyr^56^**	-	B:Lys^124^	**A:Asp^122^, B:Asp^122^**
**6**	PD-L1-JQT inhibitor	-	A:Ala^18^, A:Phe^19^, A:Ile^54^, A:Val^55^, A:Tyr^56^, A:Met^115^, A:Ile^116^, A:Ala^121^ A:Tyr^123^, B:Ile^54^, B:Tyr^56^, B:Val^68^, B:Met^115^, B:Ile^116^, B:Ala^121^, B:Tyr^123^	A:Thr^20^, A:Gln^66^, A:Ser^117^, B:Asn^63^, B:Gln^66^, B:Ser^117^	A:Lys^124^, B:Tyr^56^, *A:Lys^124^	A:Lys^124^	A:Lys^124,^ A:Arg^125^	A:Asp^122^, B:Asp^61^, B:Asp^122^

Symbol asterisk (*) symbol represents the residues showing *π-Cation interactions. Residues in ‘bold text’ are the same as exhibited by the JQT inhibitor.

**Table 3:** Intermolecular interactions profiles for the extracted last poses from 100 ns MD simulation trajectories at 4 Å distance around the docked conformations of ligands with PD-L1.

S. No.	Complex	H-Bond	Hydrophobic	Polar	π-π/*π-Cation	Salt Bridge	Positive	Negative
1	PD-L1-Neoenactin B1	**B:Tyr^56^,** B:Asp^61^	**A:Ile^54^, A:Val^55^, A:Tyr^56^, A:Met^115^,** **A:Ile^116^, A:Ala^121^, A:Tyr^123^, B:Ile^54^, B:Tyr^56^,** B:Val^76^, **B:Met^115^, B:Ile^116^, B:Ala^121^, B:Tyr^123^**	**A:Ser^117^, B:Asn^63^, B:Gln^66^, B:Ser^117^**	-	**B:Asp^61^**	B:Lys^62^	**A:Asp^122^, B:Asp^61^, B:Asp^122^**
2	PD-L1-Actinofuranone I	**A:Asp^122^, B:Tyr^56^**	**A:Ile^54^, A:Val^55^, A:Tyr^56^, A:Met^115^, A:Ile^116^, A:Ala^121^, A:Tyr^123^, B: Ile^54^, B:Tyr^56^, B:Val^68^,** B:Val^76^, **B:Met^115^,** **B:Ile^116^, B:Ala^121^, B:Tyr^123^**	**A:Ser^117^,** B:Asn^63^, **B:Ser^117^**	-	-	**A:Lys^124,^** A:Arg^125^	**A:Asp^122^,** B:Glu^58^, **B:Asp^122^**
3	PD-L1-Cosmosporin A	**A:Tyr^123^, B:Asp^122^**	**A:Ile^54^, A:Tyr^56^, A:Met^115^, A:Ile^116^, A:Ala^121^, A:Tyr^123^, B: Ile^54^, B:Val^55^, B:Tyr^56^, B:Val^68^, B:Met^115^, B:Ile^116^, B:Ala^121^, B:Tyr^123^**	**A:Ser^117^,** **B:Gln^66^** **,** **B:Ser^117^**	-	-	**A:Lys^124^,** A:Arg^125^	**A:Asp^122^,** B:Glu^58^, **B:Asp^122^**
4	PD-L1-Ganocapenoid A	**A:Tyr^56^, B:Gln^66^**	**A:Ile^54^, A:Tyr^56^,** A:Val^76^, **A:Met^115^,** **A:Ala^121^, A:Tyr^123^, B:Ile^54^, B:Val^55^, B:Tyr^56^, B:Val^68^,** B:Val^76^, **B:Met^115^, B:Ile^116^, B:Ala^121^, B:Tyr^123^**	A:Asn^63^, **A:Gln^66^,** **B:Gln^66^, B:Ser^117^**	-	-	B:Arg^113^	A:Glu^58^, **A:Asp^122^,** **B:Asp^122^**
5	PD-L1-3-[3-hydroxy-4-(3-methylbut-2-enyl)phenyl]-5-(4-hydroxybenzyl)-4-methyldihydrofuran-2(3H)-one	**B:Met^115^**	**A:Ile^54^, A:Tyr^56^, A:Met^115^, A:Ile^116^,** **A:Ala^121^, A:Tyr^123^, B:Ile^54^,** B:Val^55^, **B:Tyr^56^, B:Val^68^, B:Met^115^, B:Ile^116^, B:Ala^121^, B:Tyr^123^**	**A:Gln^66^, A:Ser^117^,** **B:Gln^66^, B:Ser^117^**	A:Tyr^56^ B:Tyr^123^	-	-	**A:Asp^122^, B:Asp^122^**
6	PD-L1-JQT inhibitor	-	**A:Ile^54^, A:Tyr^56^, A:Met^115^, A:Ile^116^, A:Ala^121^, A:Tyr^123^, B:Ile^54^, B:Tyr^56^, B:Val^68^,** B:Val^76^, **B:Met^115^, B:Ile^116^, B:Ala^121^, B:Tyr^123^**	A:Thr^20^, **A:Gln^66^,** **A:Ser^117^,B:Gln^66^,** **B:Ser^117^**	B:Tyr^56^, *A:Arg^125^	A:Lys^124^	**A:Lys^124^,** A:Arg^125^	**A:Asp^122^,** **B:Asp^122^**

Symbol asterisk (*) symbol represents the residues showing *π-Cation interactions. Residues in ‘bold text’ are the same as exhibited by the docked complex.

## Data Availability

The datasets used and/or analyzed during the current study are available from the corresponding author on reasonable request.

## Supplementary Materials

The following supporting information can be downloaded at: https://www.mdpi.com/article/10.3390/life12050659/s1, Table S1: ADMET prediction for the screened five natural compounds as PD-L1 inhibitor, Table S2: Calculated net binding free energy for the selected docked poses of PD-L1-natural compounds snap shots from the last 10 ns interval of 100 ns MD simulation, Figure S1: (a) 3D and (b) 2D interaction poses of the reference complex, i.e., PD-L1-JQT inhibitor. In 2D interaction maps, green line (π-π stacking), red line (π-cation stacking), red-violet line (salt-bridge), red color residues (negative), violet color residues (positive), green color residues (hydrophobic), and blue color residues (polar) exhibits the intermolecular interactions at a radius of 4 Å around the ligand in the respective docked complexes, Figure S2: (a) RMSD and (b) RMSF plots for the apo-PD-L1 receptor extracted from 100 ns MD simulation, Figure S3: RMSF plot generated for the PD-L1 docked with selected natural compounds, i.e., (a) Neoenactin B1, (b) Actinofuranone I, (c) Cosmosporin A, (d) Ganocapenoid A, (e) 3-[3-hydroxy-4-(3-methylbut-2-enyl)phenyl]-5-(4-hydroxybenzyl)-4-methyldihydrofuran-2(3H)-one, and (f) JQT inhibitor, Figure S4: RMSF plot of the selected compounds; (a) Neoenactin B1, (b) Actinofuranone I, (c) Cosmosporin A, (d) Ganocapenoid A, (e) 3-[3-hydroxy-4-(3-methylbut-2-enyl)phenyl]-5-(4-hydroxybenzyl)-4-methyldihydrofuran-2(3H)-one, and (f) JQT inhibitor, Figure S5: Schematic representation for interaction profile of PDL1 and selected natural compounds, i.e., (a) Neoenactin B1 (b) Actinofuranone I (c) Cosmosporin A, (d) Ganocapenoid A (e) 3-[3-hydroxy-4-(3-methylbut-2-enyl)phenyl]-5-(4-hydroxybenzyl)-4-methyldihydrofuran-2(3H)-one, and (f) PD-L1-JQT inhibitor, extracted at 30% of total 100 ns MD simulation interval.
