# Supplementary material for: Computational Investigations on the Natural Small Molecule as an Inhibitor of Programmed Death Ligand 1 for Cancer Immunotherapy
Source: Life (Basel). 2022 Apr 29;12(5):659. doi: 10.3390/life12050659 (PMC9145275; doi:10.3390/life12050659)
Supplement: Supplementary file 1 [file life-12-00659-s001.zip › life-1687328-supplementary.pdf]

life

Article

Computational Investigations on the Natural Small Molecule as an Inhibitor of Programmed Death Ligand 1 for Cancer Immunotherapy

Geethu S Kumar [1,2], Mahmoud Moustafa [3,4], Amaresh Kumar Sahoo [5,*], Petr Malý [6,*] and Shiv Bharadwaj [6,*]

[1] Department of Life Science, School of Basic Science and Research, Sharda University, Greater Noida 201310, Uttar Pradesh, India; geethusaji1440@gmail.com
[2] Center for Bioinformatics, Computational and Systems Biology, Pathfinder Research and Training Foundation, Greater Noida 201308, India
[3] Department of Biology, Faculty of Science, King Khalid University, Abha 62529, Saudi Arabia; mfmostfa@kku.edu.sa
[4] Department of Botany and Microbiology, Faculty of Science, South Valley University, Qena 83523, Egypt
[5] Department of Applied Sciences, Indian Institute of Information Technology Allahabad, Allahabad 211015, Uttar Pradesh, India
[6] Laboratory of Ligand Engineering, Institute of Biotechnology of the Czech Academy of Sciences, v.v.i., BIOCEV Research Center, Vestec 25250, Czech Republic
* Correspondence: asahoo@iiita.ac.in (A.K.S.); petr.maly@ibt.cas.cz (P.M.); shiv.bharadwaj@ibt.cas.cz (S.B.)

Supplementary Materials:

S1. Results and discussion

S1.1. Virtual screening and ADMET analysis

Table S1. ADMET prediction for the screened five natural compounds as PD-L1 inhibitor.

Characteristics	Neoenactin B1	Actinofuranone I	Cosmosporin A	Ganocapenoid A	3-[3-hydroxy-4-(3-methylbut-2-enyl)phenyl] -5-(4-hydroxybenzyl)-4-methyldihydrofuran-2(3H)-one
Heavy atoms	27	30	26	27	27
Aromatic heavy atoms	0	0	6	6	12
Fraction Csp3	0.85	0.61	0.55	0.48	0.35
Rotatable bonds	18	11	9	9	5
H-bond acceptors	6	7	4	6	4
H-bond donors	3	5	4	4	2
MR	106.35	115.84	109.13	103.3	106.87
TPSA	120.93	127.45	80.92	107.22	66.76
iLOGP	3.17	3.54	4.06	2.63	3.18
XLOGP3	1.42	2.24	4.93	2.71	5.55
WLOGP	2.61	2.04	4.53	2.8	4.49
MLOGP	1.16	0.44	3.19	1.66	3.58
Silicos-IT Log P	3.3	3.74	4.97	3.5	4.81
Consensus Log P	2.33	2.4	4.34	2.66	4.32
ESOL Log S	-1.94	-3.16	-4.77	-3.45	-5.61
ESOL Solubility (mg/ml)	4.41E+00	2.96E-01	6.15E-03	1.33E-01	9.05E-04
ESOL Solubility (mol/l)	1.14E-02	6.96E-04	1.70E-05	3.53E-04	2.47E-06
ESOL Class	Very soluble	Soluble	Moderately soluble	Soluble	Moderately soluble
Ali Log S	-3.56	-4.55	-6.37	-4.61	-6.71
Ali Solubility (mg/ml)	1.05E-01	1.19E-02	1.56E-04	9.14E-03	7.11E-05
Ali Solubility (mol/l)	2.73E-04	2.80E-05	4.30E-07	2.43E-05	1.94E-07
Ali Class	Soluble	Moderately soluble	Poorly soluble	Moderately soluble	Poorly soluble
Silicos-IT LogSw	-3.62	-1.62	-4.29	-3.31	-5.82
Silicos-IT Solubility (mg/ml)	9.25E-02	1.01E+01	1.88E-02	1.84E-01	5.53E-04
Silicos-IT Solubility (mol/l)	2.39E-04	2.39E-02	5.18E-05	4.88E-04	1.51E-06
Silicos-IT class	Soluble	Soluble	Moderately soluble	Soluble	Moderately soluble
GI absorption	High	High	High	High	High
BBB permeant	No	No	No	No	Yes
Pgp substrate	Yes	Yes	Yes	Yes	No
CYP1A2 inhibitor	No	No	Yes	No	Yes
CYP2C19 inhibitor	No	No	No	No	Yes
CYP2C9 inhibitor	No	No	No	No	Yes
CYP2D6 inhibitor	No	No	No	No	No
CYP3A4 inhibitor	No	No	Yes	No	Yes
log Kp (cm/s)	-7.65	-7.3	-5.01	-6.67	-4.59
Lipinski violations	0	0	0	0	0
Ghose violations	0	0	0	0	0
Veber violations	1	1	0	0	0
Egan violations	0	0	0	0	0
Muegge violations	1	0	0	0	1
Bioavailability Score	0.55	0.56	0.55	0.55	0.55
PAINS alerts	0	0	0	0	0
Brenk alerts	2	2	1	2	1
Leadlikeness violations	2	2	3	2	2
Synthetic Accessibility	4.12	6.06	3.8	4.07	4.07

S1.2 Molecular contact analysis

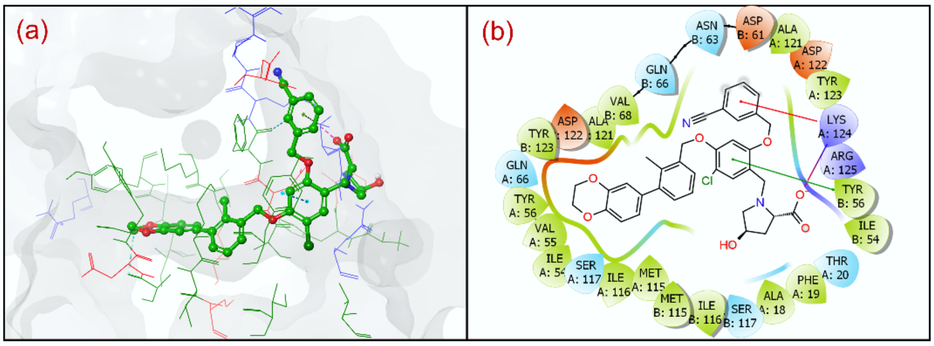

Figure S1. (a) 3D and (b) 2D interaction poses of the reference complex, i.e., PD-L1-JQT inhibitor. In 2D interaction maps, green line (π-π stacking), red line (π-cation stacking), red-violet line (salt-bridge), red color residues (negative), violet color residues (positive), green color residues (hydrophobic), and blue color residues (polar) exhibits the intermolecular interactions at a radius of 4 Å around the ligand in the respective docked complexes.

S1.2. Molecular Dynamics simulation analysis

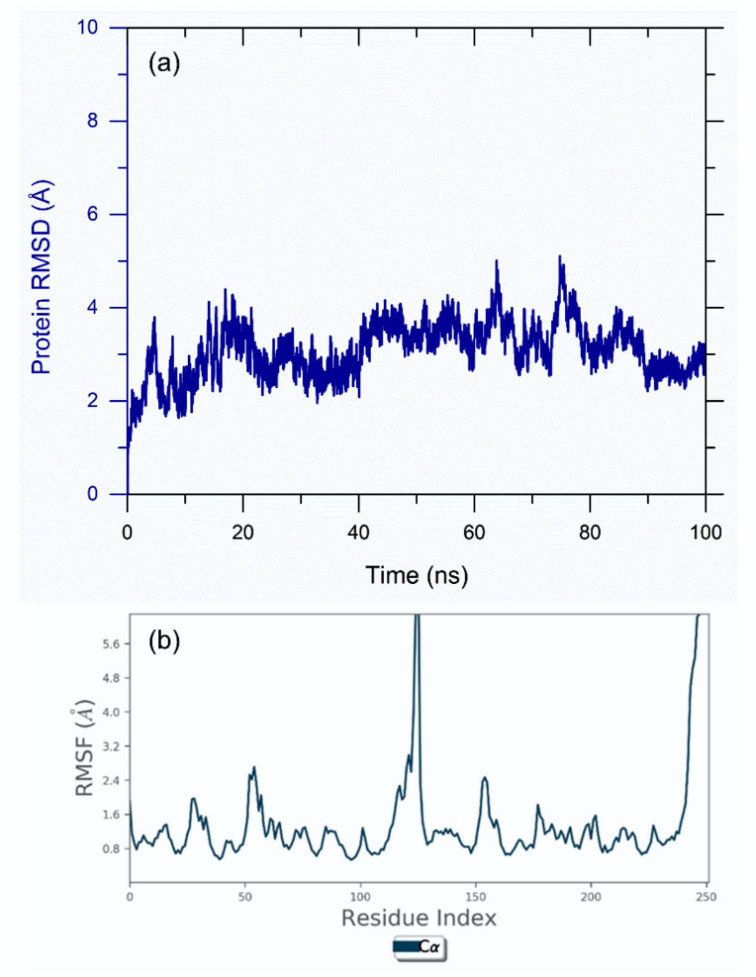

Figure S2. (a) RMSD and (b) RMSF plots for the apo-PD-L1 receptor extracted from 100 ns MD simulation.

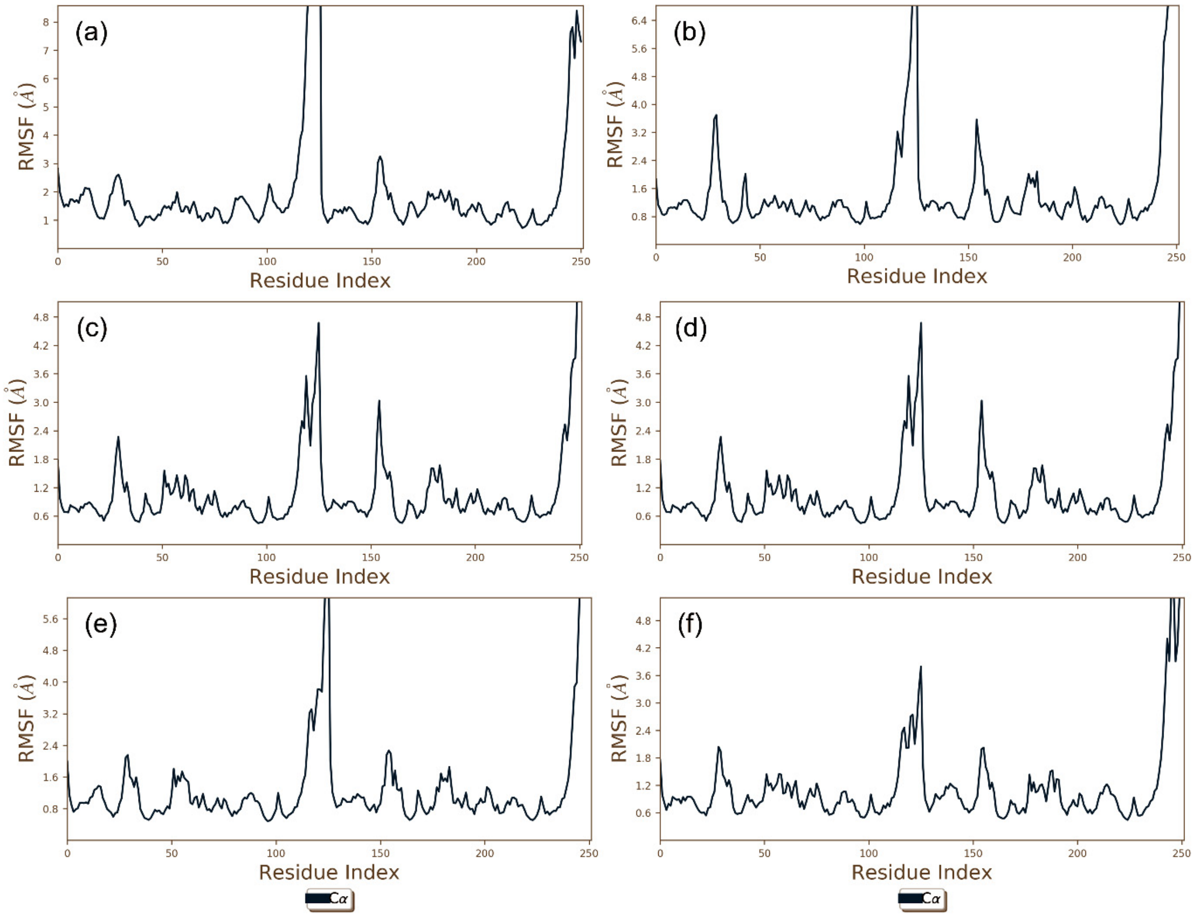

Figure S3. RMSF plot generated for the PD-L1 docked with selected natural compounds, i.e. (a) Neoenactin B1, (b) Actinofuranone I, (c) Cosmosporin A, (d) Ganocapenoid A, (e) 3-[3-hydroxy-4-(3-methylbut-2-enyl)phenyl]-5-(4-hydroxybenzyl)-4-methyldihydrofuran-2(3H)-one, and (f) JQT inhibitor.

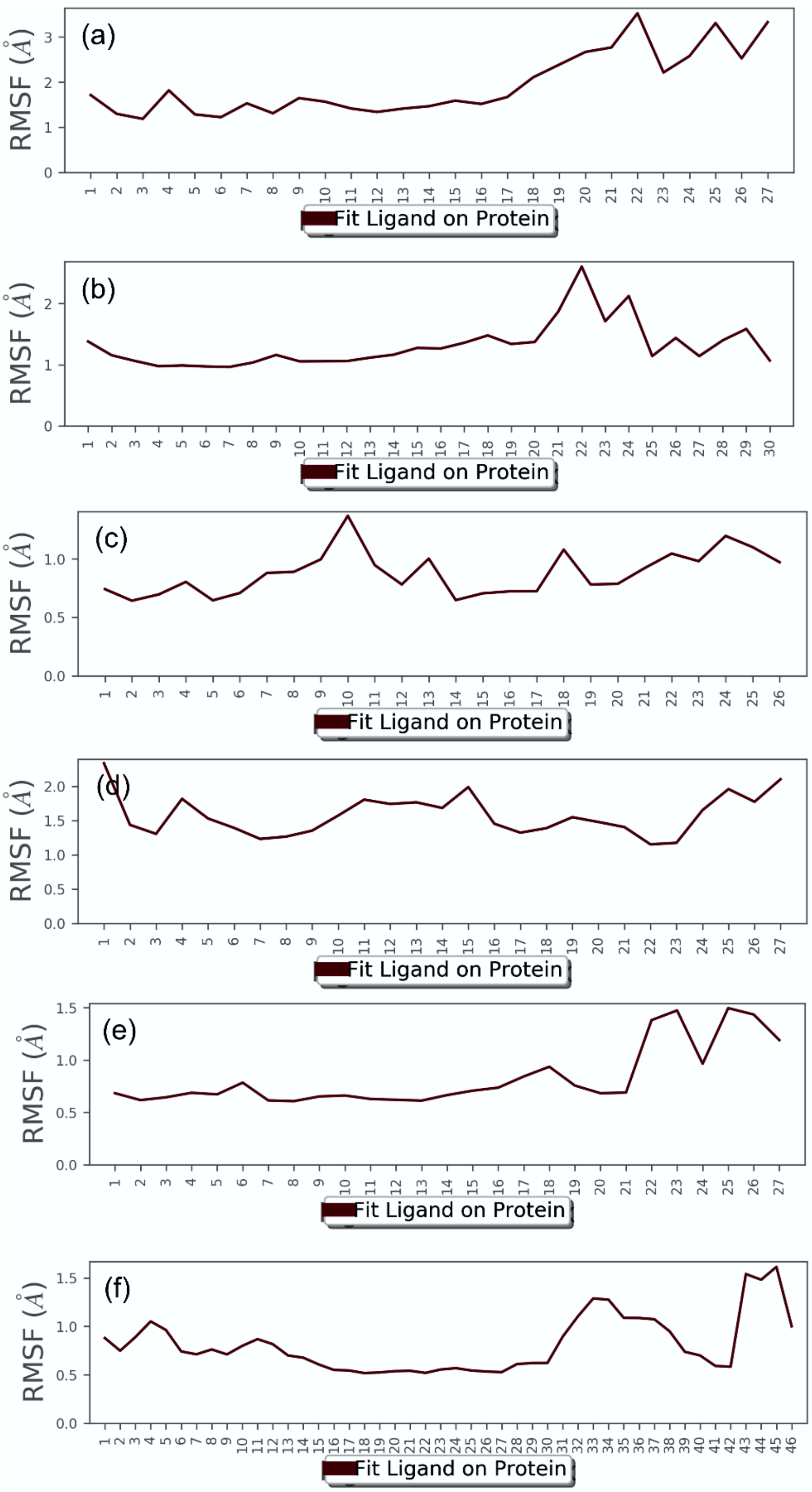

Figure S4. RMSF plot of the selected compounds; (a) Neoenactin B1, (b) Actinofuranone I, (c) Cosmosporin A, (d) Ganocapenoid A, (e) 3-[3-hydroxy-4-(3-methylbut-2-enyl)phenyl]-5-(4-hydroxybenzyl)-4-methyldihydrofuran-2(3H)-one, and (f) JQT inhibitor.

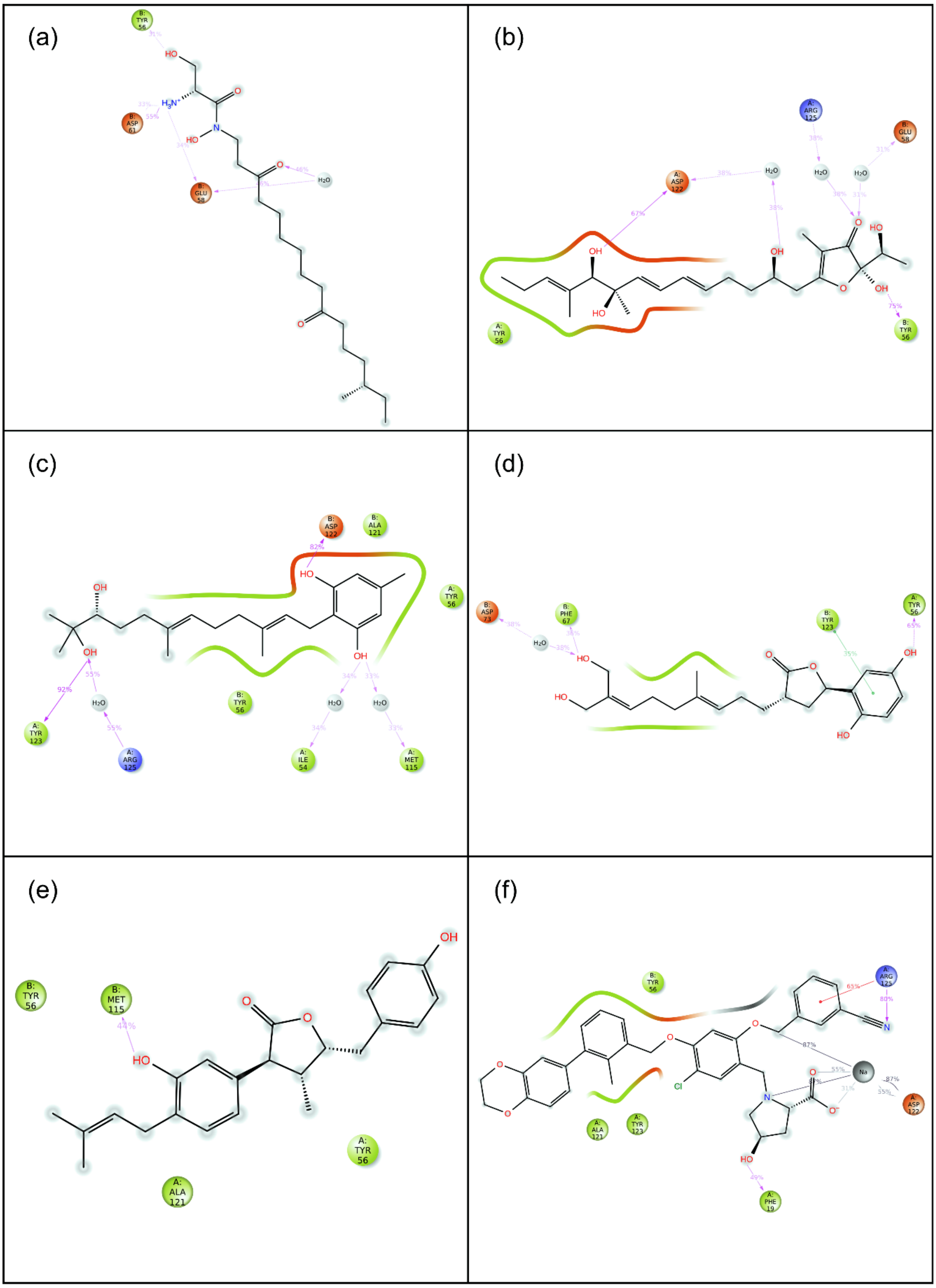

Figure S5. Schematic representation for interaction profile of PDL1 and selected natural compounds, i.e., (a) Neoenactin B1 (b) Actinofuranone I (c) Cosmosporin A, (d)Ganocapenoid A (e) 3-[3-hydroxy-4-(3-methylbut-2-enyl)phenyl]-5-(4-hydroxybenzyl)-4-methyldihydrofuran-2(3H)-one, and (f) PD-L1-JQT inhibitor, extracted at 30% of total 100 ns MD simulation interval.

S1.3. Binding free energy analysis

Table S2. Calculated net binding free energy for the selected docked poses of PD-L1-natural compounds snap shots from the last 10 ns interval of 100 ns MD simulation.

**Energy (kcal/mol)**	**PD-L1–Neoenactin B1**		**PD-L1–Actinofuranone I**		**PD-L1–Cosmosporin A**		**PD-L1–Ganocapenoid A**		**PD-L1–3-[3-hydroxy -4-(3-methylbut-2-enyl)phenyl]-5-(4-hydroxy benzyl)-4-methyl dihydrofuran-2(3H)-one**		**PD-L1–JQT inhibitor**	
	**Docked Pose**	**MD trajectory**	**Docked Pose**	**MD trajectory**	**Docked Pose**	**MD trajectory**	**Docked Pose**	**MD trajectory**	**Docked Pose**	**MD trajectory**	**Docked Pose**	**MD trajectory**
ΔG_{Bind}	-79.45	-73.55 ± 7.62	-71.97	-81.07 ± 6.41	-67.43	-83.96 ± 4.51	-66.92	-84.56 ± 6.36	-64.78	-85.35 ± 5.56	-96.7	-112.91 ± 8.33
$\Delta G_{Bind\ Coulomb}$	-65.69	-36.62 ± 13.94	-25.08	-24.19 ± 5.52	-22.82	-18.41 ± 3.49	-25	-23.99 ± 7.12	-12.17	-14.99 ± 4.52	32.03	-6.76 ± 19.50
$\Delta G_{Bind\ Covalent}$	9.79	5.36 ± 2.62	4.96	3.63 ± 1.38	5.05	3.88 ± 1.28	12.32	4.59 ± 1.77	8.41	3.37 ± 1.50	4.35	2.24 ± 1.62
$\Delta G_{Bind\ Hbond}$	-3.96	-2.02 ± 0.96	-2.33	-1.93 ± 0.54	-3.21	-1.44 ± 0.43	-1.35	-1.85 ± 0.45	-1.49	-0.79 ± 0.35	-1.57	-0.91 ± 0.42
$\Delta G_{Bind\ Lipo}$	-26.11	-24.46 ± 1.81	-30.73	-29.61 ± 1.43	-30.41	-31.18 ± 1.36	-31.64	-31.28 ± 1.76	-38.69	-35.06 ± 1.57	-47.09	-43.84 ± 1.85
$\Delta G_{Bind\ Packing}$	0 ± 0	0 ± 0	0 ± 0	0 ± 0	-0.19	-0.54 ± 0.35	-2.11	-3.52 ± 0.58	-2.37	-2.54 ± 0.70	-4.03	-5.29 ± 0.72
$\Delta G_{Bind\ Solv\ GB}$	52.18	41.38 ± 12.09	37.43	33.13 ± 3.03	37.95	24.55 ± 2.26	32.65	31.02 ± 3.85	35.52	27.77 ± 2.67	11.29	22.01 ± 13.47
$\Delta G_{Bind\ vdW}$	-45.66	-57.18 ± 4.54	-56.23	-62.09 ± 2.79	-53.79	-60.81 ± 2.58	-53.33	-59.52 ± 3.04	-55.98	-63.09 ± 2.96	-91.67	-80.36 ± 3.26
**Lig Strain Energy**	13.859	5.55 ± 2.66	11.27	5.24 ± 1.37	8.252	4.82 ± 1.70	19.88	6.03 ± 2.33	12.572	4.47 ± 1.45	7.179	4.78 ± 1.62
